# Simultaneous external and internal marking of *Triatoma sordida* nymphs: trace element efficacy and microgeographic dispersal in a peridomestic Brazilian Cerrado rural household

**DOI:** 10.1186/s13071-022-05451-z

**Published:** 2022-09-14

**Authors:** Edson Santos Dantas, Rodrigo Gurgel-Gonçalves, Rafael Maciel-de-Freitas, Fernando Araújo Monteiro

**Affiliations:** 1 Laboratório de Transmissão de Hematozoários, Instituto Oswaldo Cruz, Fundação Oswaldo Cruz (IOC/ FIOCRUZ), Rio de Janeiro, Brazil; 2 Laboratório de Parasitologia Médica e Biologia de Vetores, Área de Patologia, Faculdade de Medicina, Universidade de Brasília, Campus Universitário Darcy Ribeiro, Brasília, DF Brazil; 3 Arbovirology Department, Bernhard-Nocht Institute for Tropical Medicine, 20359 Hamburg, Germany; 4 Laboratório de Epidemiologia e Sistemática Molecular, Instituto Oswaldo Cruz, Fundação Oswaldo Cruz (IOC/ FIOCRUZ), Rio de Janeiro, Brazil

**Keywords:** Mark-release-recapture, Dispersal, *Triatoma sordida*, Nymphs, Trace element

## Abstract

**Background:**

Chagas disease (American trypanosomiasis) is an important neglected tropical illness, which has the flagellate protozoan *Trypanosoma cruzi* as etiological agent and blood-feeding insects of the Triatominae subfamily as vectors. Despite its importance for disease epidemiology, field studies targeting microgeographic dispersal of triatomines in endemic areas are rare. The ability wingless nymphs have to move (crawl) within peridomestic settings is a key component regarding the design and development of rational control strategies.

**Methods:**

We double-marked *Triatoma sordida* fourth-instar nymphs (N4) with a reliable fluorescent dye and a trace element. This new methodology allowed us to simultaneously evaluate (i) nymph dispersal and (ii) the effectiveness of copper (Cu), chromium (Cr), and cadmium (Cd) trace elements as potential new markers. In the mark-release-recapture (MRR) experiment, 390 T*. sordida* N4 were released in the peridomicile of a single rural household, 130 individuals at each of three release points, at distances of 2, 5, and 10 m from the chicken coop (CC) and 27, 32, and 35 m away from the horse corral (HC). All specimens were double marked (Cu/blue, Cr/orange, Cd/green). Recaptures occurred in two intervals: 1–3 days and 15–17 days after release.

**Results:**

Specimens were successfully recaptured at all distances up to 10 m. A total of 19, 23, and 10 specimens were able to disperse 2, 5, and 10 m, respectively, to reach the CC. No insects were recaptured at the HC. Of the three analyte/paint combinations tested, Cr/orange gave the most promising results; Cu/blue marker and Cd/green marker performed very poorly with only 4/19 and 0/10 analyte/paint ratios, respectively.

**Conclusions:**

*Triatoma sordida* N4 could cover a distance of 10 m in 17 days. This indicates that nymphs seem to have a reduced dispersal capability compared to adults. Ninety-one percent of the 22 recaptured orange-marked nymphs were still Cr positive after the 17-day period evaluated. This makes this analyte a good candidate for future investigations that will apply this marking method in MRR studies.

**Graphical Abstract:**

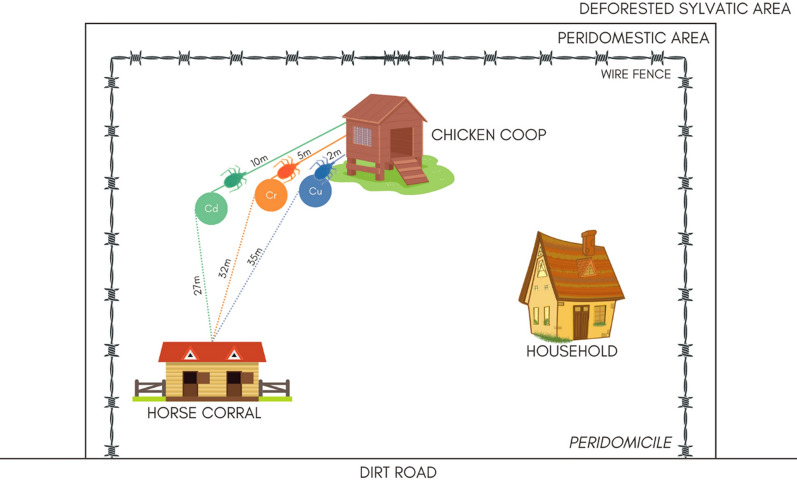

## Background

Chagas disease is still a major public health concern in Latin America [1, 2]. Caused by the flagellate *Trypanosoma cruzi* and transmitted to humans by blood-feeding insects of the Triatominae subfamily [3], the most common way to acquire the infection is through contact of the contaminated feces of the insect vector with the host’s mucous membranes [4, 5]. The disease has become an issue in nonendemic areas as well, such as the USA, Europe, and Japan, as a consequence of the immigration of infected individuals who act as blood or organ donors [6].

Despite its relevance for disease epidemiology, field studies dedicated to the biology of triatomine insects on endemic sites are rare, particularly those focusing on their movement between ecotopes. The ability to move from the sylvatic, peridomestic and domestic environments, by either flight or crawling, is a key component regarding the likelihood estimation of a given species to colonize the surroundings of human dwellings [7].

Mark, release, and recapture (MRR) is the most used technique to estimate parameters such as population size, dispersal, and survival rate in ecological studies of target species [8–11]. For triatomines, MRR has been the method of choice to evaluate flight activity and the movement among the sylvatic, peridomestic, and domestic environments [7, 12–17]. Most MRR triatomine dispersal studies have however preferably investigated the movement of adult insects, thus overlooking the potential role immature stages (also competent vectors) might play in disease transmission dynamics [18, 19].

The absence of MRR assays reporting field-derived data on nymphs might be partially due to the lack of a trustworthy, long-lasting effective marker. So far, MRR studies focused on triatomine bugs have relied mostly on topical marking methods, whose effectiveness is compromised when applied to immature stages that go through sequential molts. Reliable estimates based on MRR methods need to satisfy a number of assumptions: (i) marking should not lead to any abnormal insect behavior that would enhance the odds of being either captured or predated, (ii) sampling odds and survival rate between sampling events should remain constant, (iii) marking should persist throughout individual lifespan, and (iv) marked and unmarked individuals must comingle completely before recapture efforts begin [20]. Consequently, trace elements might represent an interesting alternative to the topical marking provided by stains and powders since it can be offered to insects via blood-feeding and are not lost with cuticle shedding [21].

Trace elements are naturally found in very low concentrations in most organisms and are usually required for maintaining their regular physiological functions. They have already been used to study life history parameters in various insect groups [21]. Medically important and blood-feeding insects have been marked with trace elements, as both larvae and adults. For instance, *Anopheles stephensis* larvae were marked by adding RbCl to the water in which they were reared, and authors have concluded that 32 ppm fosters detection in adults with minimal toxic effects [22]. Maciel-de-Freitas et al. [23] marked *Aedes albopictus* females after offering sheep blood mixed with RbCl, which resulted in a marking rate on egg batches > 80% up to 61 days. Most importantly, RbCl marking had no effect on the survival of females and their egg production compared to uninfected counterparts, allowing further estimates of dispersal in field settings [23, 24].

The possibility of using trace elements to mark Chagas disease vectors has only begun to be evaluated. For example, *Triatoma brasiliensis* marked with chromium chloride (CrCl_3_) presented a positive rate of 93% up to 119 days post-marking, whereas RbCl was effectively detected in only 2.5% of marked individuals [25]. No single trace element serves as a ubiquitous marker. For example, while RbCl was reliable for *Ae. albopictus* [23], it was ineffective for *T. brasiliensis* [25]. Additional evidence of trace element specificity in marking triatomines came with the observation that > 97% of *T. brasiliensis* individuals that were fed chromium-enriched blood were still marked 120 days after the blood meal. On the other hand, < 70% of the chromium-marked *T. pseudomaculata* insects remained positive 120 days post-marking [26].

Therefore, this article aims to answer two relevant epidemiological questions via the application of an innovative methodology. Every nymph used in this study was simultaneously double marked with a reliable fluorescent dye and a particular trace element. This experimental strategy allowed us to concomitantly evaluate (i) the dispersal capabilities of *T. sordida* fourth-instar nymphs (N4) and (ii) the persistence and effectiveness of three trace elements as potential new markers for this species.

## Methods

### Study site

The study was conducted in the peridomestic area of a rural household located in the city of Posse, Goiás state, Central Region of Brazil (14°07′24"S, 46°31′52"W; see [12] for map). The characteristics of the local vegetation are those of the Brazilian Cerrado, the second largest Brazilian biome. Most dwellings in the region are made of bricks and clay roof tiles and have peridomestic structures such as chicken coops (CC), horse corrals (HC), pigsties, and corn storage units. The predominant climate in the Cerrado Domain is seasonal, dry winter Tropical. The average annual temperature is around 22–23 °C, but during summer temperatures reach 40 °C. The house chosen for the MRR experiment had a CC and an HC as peridomestic structures (see [12] for details on sizes and building materials used). The former, where a *T. sordida* colony was present, offered refuge to eight chickens and the latter to two horses.

### Triatomine collection

Searches were carried out in 39 peridomestic structures (32 dwellings) such as CC, HC, pigsties, and corn storage units in Posse between August–September 2014 and January 2015. Insects were captured manually with metal tweezers from 9:00 a.m. until 4:00 p.m. Collected specimens were brought to an insectary located at the Laboratory of Medical Parasitology and Vector Biology of the University of Brasília (located at approximately 320 km from the city of Posse) in 50-ml Falcon tubes. Individuals were identified based on their morphological characteristics with the aid of adequate taxonomic keys identified [3], placed in appropriate cages, and kept in the insectary (mean temperature of 30 ± 2 °C, with no humidity control). The *T. sordida* collected were separated in two groups: (i) the first group was used to determine the natural absorbance of trace elements on wild specimens of *T. sordida*; (ii) the second group was used to start a laboratory colony for further MRR experiments [12].

### Trace elements on wild *T. sordida*

The first step was the election of the proper markers. For that, we determined the absorbance of 13 analytes (As, Cd, Cr, Cu, Mn, P, Pb, Rb, S, Sb, Se, Si, and Zn) in 10 field-caught *T. sordida* adults and 10 nymphs per stage from second (N2) to fifth instar (N5). This analyte panel was determined based on a combination of published papers regarding their use as trace elements to mark insects (reviewed by [21]) and low analyte concentration in field-collected insects. Each insect was dissolved by heating to 100 °C in 2 ml of 65% nitric acid [25]. Once the nitric acid had completely evaporated, we added 2 ml of distilled water, homogenized the solution, and transferred 1 ml to a plastic microtube. The exploratory analysis of trace element on wild individuals to determine the natural absorbance of the 13 chemical elements tested was done by optical emission spectrometry on an iCAP 6300 ICP OES (Thermo Scientific, Cambridge, England). Based on the results obtained, three elements (Cd, Cr, and Cu) were selected for the subsequent MRR experiment with *T. sordida* N4.

### Marking insects with trace elements and fluorescent paint for the MRR experiment

A total of 390 N4 were obtained and divided into three groups of 130 nymphs each to which one of three previously selected trace elements was offered in a blood meal (associated with its chloride form, CrCl_3_, CuCl_2_, and CdCl_2_) [25, 26]. Each blood meal consisted of a 20 ml solution with 0.010 mg l^−1^ of the trace element in 20 ml of fresh chicken blood (extracted into vacuum tubes containing heparin). The solution was offered to insects a single time, in the morning of the day of release (Day 0), for 30–40 min using an artificial feeding apparatus [27]. All nymphs had already been tagged with a fluorescent paint on their pronotum the night prior to release. This traditional and already established marking system served as a ‘gold standard’ with which trace element results could be compared.

The paint was produced after mixing a fluorescent dust with water (as detailed in [12]). This double-marking strategy was used to increase recapture rate of analyte-marked insects and thus allow for proper evaluation of trace element marking persistence in the field. External marking also provided direct estimates for nymph dispersal in field conditions. Therefore, insects internally marked with Cu, Cr, and Cd received blue, orange, and green external fluorescent markings, respectively.

### Marked nymph release

**Fig. 1:**
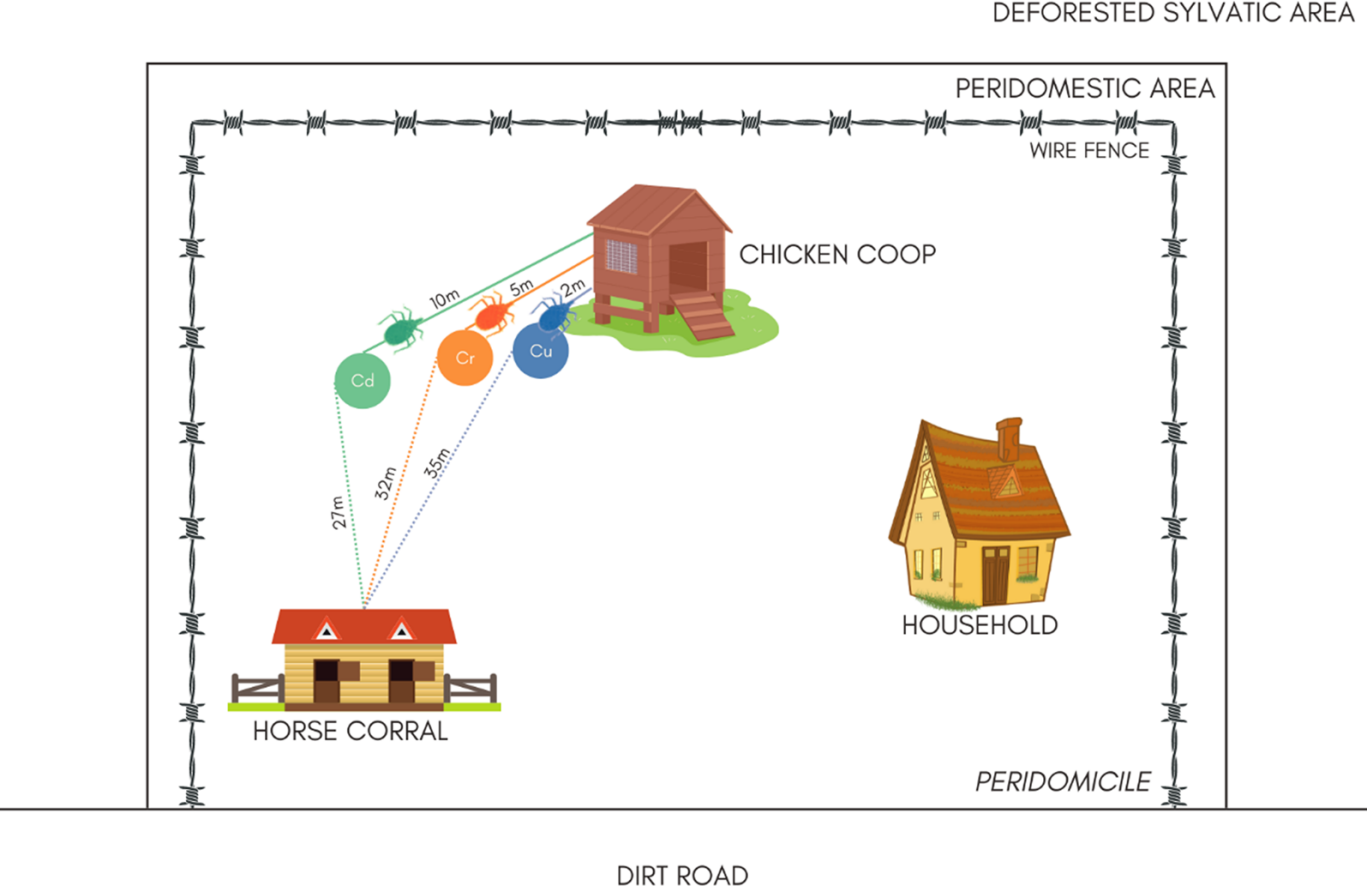
Schematic representation of the study area depicting the two peridomestic structures inspected during recaptures of *T. sordida* N4: the chicken coop and the horse corral. Also shown are release points and distances evaluated. Insects internally marked with Cu, Cr, and Cd received blue, orange, and green external fluorescent markings, respectively. Abbreviations: Cu, Cr, and Cd stand for copper, chromium, and cadmium

Due to the lack of information on the active dispersal capacity of nymphs, we decided to use a simple strategy, analogous to the one we have already successfully implemented with adult insects [12]. A total of 390 double-marked N4 were released at three points (130 specimens at each point) along a straight line at distances of 2 (Cu/blue), 5 (Cr/orange), and 10 m (Cd/green) from the CC. These points were also 35, 32, and 27 m away, respectively, from the HC (Fig. 1; [12]). Nymphs were released at dusk on Day 0.

### Marked nymph recapture

Recaptures were carried out in the mornings of the first 3 days after release and then again 2 weeks later for another 3-day period. In all recapture events both structures (CC and HC) were inspected for the presence of marked triatomine insects. The inspection was conducted by three health agents during 60 min in each recapture site. To evaluate trace element marking efficacy, all N4 found were captured and brought to the laboratory for trace element detection. As we suspected that some released N4 might suffer ecdysis and lose their external marking, we also collected all N5 we encountered.

### Trace element detection in field-caught insects

Individual specimens were dissolved through heating to 100 °C in 2 ml of 65% nitric acid [25]. Once the nitric acid had completely evaporated, we added 2 ml of distilled water to the specimen, homogenized it, and transferred 1 ml to a plastic microtube. The exploratory analysis of the trace element on wild individuals to determine the natural absorbance of the 13 chemical elements tested was done by optical emission spectrometry on the iCAP 6300 ICP OES (Thermo Scientific, Cambridge, England). More sensitive equipment (Inductively Coupled Plasma Mass Spectrometry, iCAP Qc ICP-MS model by Thermo Fisher Scientific, Bremen, Germany) was used to determine the absorbance of Cd, Cr, and Cu from field-caught individuals.

To assess which insects were positive for Cd, Cr, and Cu, we used the mean absorbance values plus three times and standard errors of the negative controls to estimate a confidence interval (α = 0.01). Unmarked adult triatomines were used as negative controls, since N4 would not turn into adults in a 15-day period and were still subject to the same ecological conditions. Wild nymphs whose absorbance values were higher than the upper limit established above were considered positive for trace element marking.

## Results

### Field survey

A total of 39 peridomestic structures on the outskirts of Posse were inspected for the presence of wild *T. sordida* insects. Twenty-five of those (64.1%) were positive, i.e. harbored at least on the specimen within their limits. A total of 541 *T. sordida* were collected (139 adults, with 80 males and 59 females, 47 N5, 149 N4, 135 N3, 61 N2, and 10 N1), with an average of 21.6 specimens per positive premise.

### Trace elements on wild triatomine insects

From the total of 541 field-caught specimens, we determined the absorbance of 13 analytes on 10 T*. sordida* adults and 10 nymphs per stage from N2 to N5 (Table 1) as a first step to select those that would become internal marking elements for the MRR experiment.

**Table 1:** Mean ± standard deviation of natural concentration (as μg/l) of 13 chemical elements in wild *Triatoma sordida* adults and nymphs (N2–N5) collected in the field site of Posse, Goiás, Brazil

Trace element	*Triatoma sordida*					Wavelength (nm)	R
	N2	N3	N4	N5	Adult		
As	< 0.01	< 0.01	< 0.01	< 0.01	< 0.01	193.759	0.9997
Cd^a^	< 0.01	< 0.01	< 0.01	< 0.01	< 0.01	226.502	0.9998
Cr^a^	< 0.01	< 0.01	0.01 ± 0.01	0.01 ± 0.01	0.03 ± 0.01	283.563	0.9998
Cu^a^	< 0.01	< 0.01	< 0.01	< 0.01	0.08 ± 0.10	324.754	0.9998
Mn	0.04 ± 0.02	0.03 ± 0.01	0.04 ± 0.02	0.03 ± 0.02	0.03 ± 0.01	257.610	0.9999
P	16.35 ± 7.12	42.7 ± 18.8	45.7 ± 31.58	58.6 ± 39.1	102.4 ± 29.04	214.914	0.9998
Pb	0.03 ± 0.01	0.03	0.04 ± 0.01	0.04 ± 0.01	0.06 ± 0.02	220.353	0.9997
Rb	0.08 ± 0.04	0.19 ± 0.09	0.12 ± 0.06	0.26 ± 0.24	0.33 ± 0.17	780.023	0.9997
S	39.7 ± 14.67	109.19 ± 49.72	107.56 ± 80.8	156.71 ± 95	247 ± 129.32	180.731	0.9997
Sb	0.01	0.01	0.01	0.01	0.01	206.833	0.9997
Se	< 0.02	< 0.02	< 0.02	< 0.02	< 0.02	196.090	0.9996
Si	0.32 ± 0.04	0.40 ± 0.08	0.33 ± 0.06	0.38 ± 0.08	0.38 ± 0.06	251.611	0.9999
Zn	0.15 ± 0.06	0.31 ± 0.21	0.56 ± 0.34	0.60 ± 0.46	2.24 ± 0.51	213.856	0.9997

The appropriate wavelength and the fit of the standard curve (R) for each chemical element tested are indicated. Ten specimens were tested per developmental stage. When all ten specimens had the same element concentration, no standard deviation was produced

^a^Denotes trace elements selected for the MRR experiment

### Choice of trace elements for MRR experiments

After assessing the natural concentration of analytes in field-caught triatomines, we selected Cd, Cr, and Cu based on their low concentration, which would facilitate the identification of analyte-blood-fed (i.e. high analyte concentration) recaptured individuals.

### MRR experiment

A total of 390 N4 *T. sordida* insects were released, 130 individuals at each of three release points, at distances of 2, 5, and 10 m from the CC and 27, 32, and 35 m away from the HC. During the course of the MRR study, a total of 100 insects were collected, 58 of them in the first three nights of collection and 42 on the second period of field capture at 15–17 days post-release. From that total, 49 were marked with fluorescent paint and represented a recapture rate of 12.56% (*n *= 49/390) (Table 2). All marked nymphs were collected in the CC (i.e. no insect was found at the HC).

**Table 2:** Number of color-marked, trace element-marked, and unmarked *T. sordida* nymphs collected during an MRR experiment conducted at Posse, GO Brazil

Recapture	Mark	Color-marked nymphs	Trace element- marked	Unmarked nymphs
Day 1	Cu/Blue	3	0	8
	Cr/Orange	15	14	
	Cd/Green	4	0	
Day 2	Cu/Blue	2	0	8
	Cr/Orange	2	2	
	Cd/Green	3	0	
Day 3	Cu/Blue	6	0	1
	Cr/Orange	4	3	
	Cd/Green	2	0	
Day 15	Cu/Blue	5	2	10
	Cr/Orange	1	1	
	Cd/Green	1	0	
Day 16	Cu/Blue	0	0	15
	Cr/Orange	0	0	
	Cd/Green	0	0	
Day 17	Cu/Blue	1	0	9
	Cr/Orange	0	0	
	Cd/Green	0	0	
Total		49	22	51

### Marking with Cu and blue paint (2 m from CC)

A total of 17 insects were captured containing a blue stain on the pronotum. Eleven of those (64.7%) were caught on the first 3 nights whereas six (35.3%) were captured during the second round of collection (Table 2). From the 17 blue-marked bugs, only 2 (11.8%) also had an increased absorbance at Cu corresponding wavelength, both captured on the first recapturing day of the second round of collection. Additionally, two *T. sordida* with no marking presented absorbance peak of Cu, showing that a total of 19 individuals were able to disperse 2 m to the CC.

### Marking with Cr and orange paint (5 m from CC)

Twenty-two orange-stained insects were captured. Twenty-one (95.5%) were caught on the first 3 nights of collection; only one was captured at the second round of collection (Table 2). Twenty (91%) of the 22 orange-marked bugs also had an increased absorbance at Cr corresponding wavelength. The two orange-marked individuals that had no peak of Cr at the respective wavelength were recaptured on 1st and 3rd days of recapture of the first round of collection. Additionally, 1 *T. sordida* with no color marking presented an absorbance peak of Cr, suggesting a total of 23 insects were able to disperse 5 m to reach the CC.

### Marking with Cd and green paint (10 m from CC)

A total of 10 insects were captured containing a green stain on their pronotum. Nine of those were caught on the first 3 nights of collection, and only 1 was captured at the second round of collection (Table 2). Of the ten green-marked bugs, none had an increased absorbance at Cd corresponding wavelength. No unmarked insect displayed a Cd peak.

### Final recapture rate

Of the 390 double-marked specimens released at the MRR experiment, 52 nymphs had at least 1 marking to confirm its identity. Therefore, a final recapture rate of 13.3% was obtained.

## Discussion

We assessed the dispersal capacity of *T. sordida* N4 in the peridomestic area of a rural house in the Brazilian Cerrado using a double-marking strategy. For that, we returned to the classical MRR methodology by using a reliable topical fluorescent dye and a trace element on its chloride form concomitantly. By doing so, we were able to demonstrate limited dispersal ability of nymphs and a varied efficacy of trace elements in providing a long-lasting marking for triatomine bugs under field conditions. Although of great epidemiological relevance, as far as we are aware, this is the first report to use trace elements on the estimation of triatomine (nymph) dispersal at the microgeographic scale in a Chagas disease-endemic area.

### Analyte efficacy

Previous laboratory-based studies regarding the marking efficacy of trace elements revealed that Cu presented high persistence in marked *T. brasiliensis* N5 since more than 97% of insects were still marked 120 days after the blood meal containing this particular trace element [26]. Of the three analyte/paint combinations tested—Cu/blue marker, Cr/orange marker, Cd/green marker—the second gave the most promising results in terms of the efficacy of the analyte used. Twenty of the 22 orange-marked nymphs had a detectable (increased) Cr emission during the 17-day period evaluated. This makes this analyte a good candidate for future studies that will apply this marking method in MRR investigations.

Curiously, it is possible that the ingestion of Cr by the *T. sordida* N4 could lead to important negative changes in their metabolism. Cr-marked *T. brasiliensis* N5 nymphs experience reduced survival rates compared to control insects, although the biochemical or molecular basis for the phenomenon remains unclear [26]. A starting point would be to look at enzymes whose function is compromised by this particular trace element. It has been shown, for example, that Cr-injected *Oxya chinensis* (Orthoptera: Acrididae) suffers alterations in the activity of antioxidant enzymes such as superoxide dismutase, catalase, and guaiacol peroxidase, increasing oxidative stress and insect mortality [28].

Cu and Cd performed very poorly with only 4/19 and 0/10 analyte/paint ratios, respectively. This suggests *T. sordida* nymphs are able to metabolize and quickly excrete these trace elements. Furthermore, taken together, those data support the hypothesis that a specific match between triatomine bug species and trace element is required to achieve a persistent marking with no effect on insect behavior and survival rates [25, 26].

### Nymph dispersal

All marked nymphs were collected in the CC (i.e. no insect was found at the HC). Nymph dispersal was limited to a maximum of 10 m in a 17-day period. Nineteen individuals were able to disperse 2 m to reach the CC; 23 insects dispersed 5 m towards the CC; 10 insects traveled 10 m to the CC. This indicates that nymphs seem to have a reduced dispersal capability compared to adults. An earlier study conducted in the same area with adult *T. sordida* revealed a much higher dispersal capability with adult insects being able to disperse up to 32 m in a comparable time frame (17 days and 15 days for this and the previous study, respectively) and under the same collection efforts [12]. It is possible that adults might have flown and thus dispersed further, ultimately reaching the HC. However, for *Triatoma infestans*, evidence indicates that adults will not spontaneously take off from ground level [29]. Alternatively, adult *T. sordida* might have dispersed further simply because of their bigger size (assuming bigger legs would translate into more efficient locomotion) compared to N4. Other factors may influence triatomine dispersion such as feeding, insecticide resistance, and energy cost. *Triatoma infestans* from Argentina dispersed mainly by walking regardless of the number of feeds, the presence of food resource, and toxicological phenotype [30]. Flight dispersal requires high energetic costs for triatomines and may delay oviposition [31] and reduce the total number of eggs produced [32]. Moreover, Lobbia and Mougabure-Cueto [30] indicated a lower dispersal capacity in blood-fed specimens, as well as in insecticide-resistant females (compared to susceptible ones), suggesting that resistance to deltamethrin is associated with adaptive costs. The finding of *T. infestans* nymphs in indoor bedrooms with blood meal contents of animals corralled 50–80 m away reveals the potential walking nymphs have to move within rural household areas of northwestern Argentina [33]. Dispersal by crawling should also be common for triatomine species with short wings and long legs, as observed in *Triatoma sherlocki*, an adaptive characteristic that may compensate for its inability to fly [34]. All evidence combined reveals the existence of variation in terms of the dispersal capacity among triatomine species and their development stages and that nymphs also play an important role in microgeographic dissemination of *Triatoma* species within and between neighboring households.

### Recapture rate

Of the 390 double-marked specimens released, 52 N4 had at least one marking to confirm their identity. Therefore, a final recapture rate of 13.3% was achieved. This is about twice as much as described by Dantas et al. [12] for adults in the same time period (7.6%). Flight could again be evoked to account for such discrepancy: adults could have flown beyond the limits of the study area resulting in a lower recovery percentage. Low recapture rates have also been reported in other studies [12, 16] and may result from insect death following experimental manipulation, post-release predation by peridomestic animals such as chickens, or ultimately be a consequence of imperfect detection due to drawbacks of the man-hour capture method [35].

### Perspectives

MRR is a powerful ecological tool that allows estimating traits such as dispersal and survival on the field. Estimating insect dispersal capacity in an endemic setting is key to improving vector control strategies by determining the distance within which wild insects are more likely to colonize the peridomestic environment. Similar MRR studies towards the investigation of dispersal capacity of other synanthropic triatomine species such as *Panstrongylus megistus*, *T. brasiliensis*, and *T. pseudomaculata* are required to provide proper evaluation of flight and walking dispersal of triatomines within peridomestic and domestic environments.

## Conclusions

Our previous studies in the Brazilian Cerrado-endemic Chagas disease area revealed that *T. sordida* adults are able to disperse up to 32 m from the release point. The N4 studied here presented a shorter dispersal ability, displacing no farther than 10 m in a 17-day period. Moreover, we showed that orange-marked nymphs were still Cr positive after the same time period. This makes this analyte a good MRR candidate for future *T. sordida* dispersal investigations. Therefore, by increasing the distance between their household and peridomestic structures such as CCs and HCs, local inhabitants could reduce the likelihood of coming into contact with *T. sordida* bugs and, consequentially, mitigate Chagas disease transmission.

## Data Availability

All data generated or analyzed during this study are included in this published article.

## Abbreviations

CC: Chicken coops
HC: Horse corral
MRR: Mark, release, and recapture
